# Clinical versus computed tomography evaluation in the diagnosis and management of deep neck infection

**DOI:** 10.1590/S1516-31802004000600006

**Published:** 2004-11-04

**Authors:** Agricio Nubiato Crespo, Carlos Takahiro Chone, Adriano Santana Fonseca, Maria Carolina Montenegro, Rodrigo Pereira, João Altemani Milani

**Keywords:** Drainage, Abscess, Infection, Neck, X-ray computed tomography scanners, Drenagem, Abscesso, Infecção, Pescoço, Tomógrafos computadorizados

## Abstract

**CONTEXT:**

Deep neck infections have high potential for severe complications and even death, if not properly managed. The difference between clinical and computed tomography findings may demonstrate that clinical evaluation alone underestimates disease extent, which may lead to conservative treatment with worse prognosis.

**OBJECTIVE:**

To compare clinical and computed tomography findings from neck spaces affected by deep neck infections and to determine the main clinical and radiological features associated with these.

**TYPE OF STUDY:**

Non-randomized retrospective study.

**SETTING:**

Department of Otolaryngology and Head and Neck, Universidade Estadual de Campinas.

**METHODS:**

Medical charts of 65 patients with deep neck infections were evaluated. Age, gender, clinical complaints, physical findings, computed tomography scan and x-ray imaging, microbiology, treatment and outcome were analyzed. All clinical signs and symptoms were evaluated and stratified in order of frequency. The frequency of neck space involvement in such infections was also assessed from the clinical and tomographic evaluation. All clinical and computed tomography findings were compared with surgical observation.

**RESULTS:**

The most frequent clinical findings were neck swelling, local pain, erythema and locally increased temperature. Physical evaluation showed that the most affected site was the submandibular triangle (49.2% of cases). However, computed tomography showed this to be the lateropharyngeal space (65% of cases) and that more than one deep cervical space was compromised in 90% of cases, as demonstrated by the extent of swelling and increased contrast signs in soft tissue.

**DISCUSSION:**

The most frequent clinical symptoms of deep cervical infections were cervical pain, increased cervical volume and fever. The important signs seen via computed tomography were increased contrast in soft neck tissues and swelling. Such examination is the most important method for correct evaluation of cervical spaces involved in infection, and thus for correct surgical drainage.

**CONCLUSIONS:**

The most frequent clinical findings were cervical mass, neck pain, local erythema and locally increased temperature. Computed tomography demonstrated that the lateropharyngeal space was the most affected neck space. More than one deep neck space was compromised in 90% of cases. Clinical evaluation underestimated the extent of deep neck infection in 70% of patients.

## RESUMO

**CONTEXTO:**

Infecções profundas do pescoço têm um potencial alto para complicações graves e morte, se não corretamente diagnosticadas e tratadas. A diferença entre resultados de avaliação clínica e tomográfica pode demonstrar que a avaliação clínica isolada subestima a extensão de doença, o que pode conduzir a tratamento conservador e a pior prognóstico.

**OBJETIVO:**

Comparar achados clínicos à tomografia computadorizada de pescoço em relação aos espaços cervicais envolvidos e determinar as características clínicas e radiológicas principais associadas com infecção de espaço profundo de pescoço.

**TIPO DE ESTUDO:**

Estudo retrospectivo não randomizado.

**LOCAL:**

Departamento de Otorrinolaringologia – Cabeça e Pescoço, Universidade Estatal de Campinas, Brasil, um centro universitário, terciário.

**MÉTODOS:**

Foi avaliado prontuário médico de 65 pacientes com infecções profundas de pescoço. Foram analisados idade, gênero, queixas clínicas, exames físicos, resultados de raios-x e tomografia computadorizada, microbiologia, tratamento e resultados. Foram avaliados os sinais clínicos e sintomas, estratificados em ordem de freqüência. A freqüência de espaços cervicais profundos envolvidos nesta infecção também foram avaliados clínico e tomograficamente. Todos resultados clínicos e tomográficos foram comparados com a observação cirúrgica em relação aos espaços cervicais afetados por infecção.

**RESULTADOS:**

Os resultados clínicos mais freqüentes foram inchaço cervical, dor local, eritema cutâneo local e aumento localizado de temperatura. O local mais afetado de acordo com a avaliação física foi o triângulo de submandibular (49,2%), mas, à tomografia computadorizada cervical, foi o espaço látero-faríngeo (65%). Mais de um espaço cervical profundo foi acometido, de acordo com a tomografia computadorizada cervical, em 90% dos pacientes, como demonstrado pela extensão do edema e aumento de captação de tecidos moles, e em geral apenas um espaço à avaliação clínica isolada.

**DISCUSSÃO:**

Os sintomas clínicos mais freqüentes das infecções cervicais profundas foram dor cervical, aumento de volume cervical e febre. Sinais importantes da tomografia computadorizada, para avaliação desta infecção, foram aumento de captação de contraste em tecidos moles do pescoço e edema. O espaço profundo do pescoço mais afetado pela infecção foi o laterofaríngeo, pela tomografia computadorizada do pescoço. O espaço submandibular foi o mais freqüente, ao exame físico, mas foi o segundo mais freqüente, de acordo com a tomografia computadorizada do pescoço, uma vez que o láterofaríngeo é um espaço difícil de ser examinado. Este exame é o mais importante para avaliação correta dos espaços cervicais envolvidos para a sua correta drenagem cirúrgica.

**CONCLUSÕES:**

Os achados clínicos mais freqüentes foram massa cervical, dor de pescoço, eritema de pele local e aumento de temperatura local. Avaliação com tomografia computadorizada cervical, demonstrou o espaço láterofaríngeo como o espaço cervical mais afetado. Mais de um espaço profundo de pescoço esteve acometido em 90% dos pacientes à tomografia computadorizada cervical. Avaliação clínica subestima a extensão de infecção profunda do pescoço em 70% de pacientes.

## INTRODUCTION

Deep neck infections have been known and described since the second century. Nowadays, however, with antibiotics and improvements in mouth care and hygiene, their incidence and severity have decreased significantly.^1-3^

The deep cervical fascia is divided into three layers: superficial, middle and deep. The superficial layer of the deep cervical fascia includes the sternocleidomastoid and trapezium muscles and the parotid and submandibular salivary glands. The medium layer includes the prelaryngeal muscles, thyroid gland, esophagus and trachea. It extends from the hyoid, superiorly, to the mediastinum, inferiorly. The deep layer is divided into two parts: the alar fascia and prevertebral fascia. The prevertebral fascia is adjacent to the cervical vertebral bodies and runs from the skull base down to the coccyx. The alar fascia is immediately anterior to the prevertebral, but reaches only the second thoracic vertebra. All three layers of the deep cervical fascia become part of the carotid space, through which the major vessels of the neck pass. Infections located in the carotid, retropharyngeal and paravertebral spaces could rapidly extend to the thorax.^1,2,4-18^

The initial sources of infection are usually the result of dental manipulation and acute bacterial tonsillitis. However, there are many predisposing factors influencing this process and modifying its outcome, which may result in death.^2,19-22^ This kind of infection has mixed flora, mostly from the oral cavity. These become pathogenic once natural barriers lose their integrity, as in acute tonsillitis, dental abscesses and foreign body trauma.^3,8,10,11,13,16,23-30^ The symptoms are presented in many different ways and may not be correlated with extent or severity of disease. In the literature, when complications occur, mortality may range from 40 up to 50%,^1,2,5,6,8-11,14-17,31,32^ usually secondary to mediastinal extension. The treatment of choice is intravenous antibiotics and surgical drainage. There are some reports referring to a more conservative approach, such as puncture and aspiration instead of surgical drainage of the abscesses.^33-36^ Full attention must be directed to the upper airway, and a tracheotomy procedure is sometimes necessary to assure proper ventilation for the patient.^3,10,16,31-33,37-40^

Clinical evaluation alone underestimates the extent of deep neck infections, which may lead to conservative treatment with worse prognosis. Consequently, computed tomography scan imaging takes on importance for the correct evaluation of such infections.

This study reviews the data available from deep neck infections treated at the Department of Otolaryngology and Head and Neck, Universidade Estadual de Campinas, over the last 15 years, with an analysis of the risk factors, signs and symptoms, computed tomography scan imaging, treatment, complications and outcome. A comparison is established between clinical and computed tomography scan findings in relation to the neck spaces involved in deep neck infections.

## METHODS

A retrospective evaluation was made using the medical charts of 65 patients with deep neck infections who were diagnosed and treated at the Department of Otolaryngology and Head and Neck, Universidade Estadual de Campinas, from January 1986 to June 2000. Age, gender, clinical complaints, physical findings, chest x-ray and computed tomography scan imaging results, microbiology, treatment and outcome were analyzed. Patients with a diagnosis of peritonsillar abscess, or for whom no computed tomography scan of the neck had been made prior to surgical drainage, were excluded from the study.

The clinical signs and tomographic findings for neck spaces affected by infection were compared after early radical treatment of the deep neck infection. All clinical signs and symptoms were also evaluated and stratified in order of frequency. The frequency with which different neck spaces were involved in such infection was also evaluated in clinical and tomographic evaluations. All clinical and computed tomography findings were compared with surgical observation in relation to neck spaces affected by infection. The outcome evaluation was carried out to verify the efficiency of radical drainage techniques. Such techniques consisted of drainage based on tomographic findings, rather than clinical findings, with dissection of all cervical spaces affected by disease or swelling.

## RESULTS

Males predominated among the patients with deep neck infections (70.8%). Higher incidence of this infection was found among patients aged between 20 to 40 years (43%). The patients’ complaints are presented in Table 1. The time elapsed between the onset of symptoms and the first medical evaluation ranged from 1 to 17 days, with an average of 5 days. Table 1 also presents the most prevalent conditions that probably elicited these complaints. Four patients (6.1%) presented with diabetes and four with acquired immunodeficiency syndrome (6.1%). Thirty-one patients (47.7%) were treated with oral antibiotics prior to their first visit to our institution, and oral penicillin was the prescribed drug in 90% of these cases. Two patients had been submitted to surgical drainage and four to drainage by puncture elsewhere.

**Table 1:** Characteristics observed in 65 patients with deep neck infection, according to their frequency

Symptoms	Frequency (%)
Pain	89.2
Neck mass	87.7
Fever	75.4
Odynophagia	63.1
Dysphagia	47.7
Signs	Frequency (%)
Neck swelling	84.6
Localized pain	76.9
Local erythema	66.7
Localized increase in temperature	55.4
Neck spaces affected: via physical evaluation	Frequency (%)
Submandibular	49.2
Lateropharyngeal	27.7
Retropharyngeal	21.5
Submental	16
Anterior	16
Neck spaces affected: via computed tomography scan	Frequency (%)
Lateropharyngeal	65.0
Submandibular	60.0
Retropharyngeal	25.0
Submental	27.5
Etiology	Frequency (%)
Dental manipulation	43.0
Pharyngotonsillites	40.0
Foreign bodies	7.0
Trauma to aerodigestive tract	3.4
Bacterial Agent	Frequency (%)
*Streptococcus viridans*	22.2
*Staphylococcus aureus*	20.0
*Streptococcus pyogenes*	15.0
*Pseudomonas*	6.0
*Anaerobes*	6.0

The features of physical signs are presented in Table 1, in order of frequency. Table 1 also presents the sites affected, as seen via physical examination. All patients were evaluated using computed tomography scan of the neck (61.5%). The neck spaces involved, as seen via image analysis of the computed tomography scan, are shown in Table 1. Two or more neck spaces with swelling were observed via computed tomography scan in 90% of the patients, even though physical examination indicated that only one space was suggestive of involvement in 80% of the patients. Clinical examination underestimated the correct extent of disease, in relation to affected neck spaces, in 70% of the patients, with the demonstration of only one space clinically and low identification of the lateropharyngeal space in comparison with the results from computed tomography scan. Physical examination correctly evaluated the affected spaces in 30% of patients, correctly showing that one space was affected in 10% of the cases and two in 20%. Swelling and increased contrast uptake in soft neck tissues were the most important signs of deep neck infection observed via computed tomography scan. Chest X-ray was done on 72.3% of patients and was normal for 91.5% of them. An enlarged mediastinum was seen in two patients (4.2%), pleural effusion in one (2.1%) and displacement of esophagus in another (2.1%).

All patients were treated with intravenous antibiotics and surgical drainage. The most frequently used antibiotics were crystalline penicillin (72.3%) and clindamycin (23%), over a period of up to 45 days (average of 10 days). Twenty-nine patients (44.6%) completed the treatment with oral antibiotics after discharge from hospital. Surgical drainage was performed immediately after admission and complete evaluation, and thus on average five days after the onset of symptoms (range: 1 to 28 days). Four patients (6.1%) were submitted to unsuccessful puncture and aspiration prior to their arrival at our hospital. Three patients (4.6%) needed surgical debridement of cervical fascia, because of suspected necrotizing fasciitis. For six patients, tracheotomy was performed under local anesthesia and prior to surgical drainage, to assure airway patency.

Complications occurred in 17 patients (26.1%). Seven patients developed head and neck sequelae in the form of large and non-esthetic scar tissue. Mediastinitis and pleural effusion were present in one case each. Six patients developed septicemia and two had other minor metabolic disturbances such as bowel distension. Sixty patients (92.3%) were successfully treated. Five patients (7.7%) died, one of them due to immunity-suppressing illness (AIDS), one due to metabolic disturbance and diabetic ketoacidosis and three due to septicemia.

Tissue, or exsudate when present, was sent for culturing from 45 patients (69.2%), and was negative in 12 cases (26.6%). A single bacterial agent was identified in 40% of the cases and multiple bacteria in 33.3%. Streptococcus viridans was the most prevalent agent (22.2%) (Table 1).

## DISCUSSION

Deep neck infections are not so frequent nowadays.^1-3^ Their incidence and lethality used to be high, before the advent of antibiotics. But even today they have high potential for severe complications and even death, if not properly managed. Dental manipulation and oropharyngeal infections are the major causal factors of deep neck infection.^3,6-8,10,11,13,16,21,22,24-29,32,39-42^ This was also observed in the present study, in which previous histories of dental manipulation were identified in 43% of the patients and recent oropharyngeal infection in 40%. The proximity of dental roots to the submandibular or sublingual spaces and the presence of fascia with loose connective tissue surrounding the pharyngeal muscles could explain both situations.

According to the literature, immunity-debilitating diseases play an important role in the development of deep neck infection but in the present study, only 13 patients (20%) had a history of systemic disease affecting their immunological response. Diabetes mellitus (6.1%) and acquired immunodeficiency syndrome (6.1%) were the most prevalent ones. Diabetes mellitus is well known for causing host immunodeficiency and, like acquired immunodeficiency syndrome, it has progressive prevalence.^1,20,22^ These two conditions are partially responsible for the recent increase in complications from deep neck infection.^1,20,22^

The high morbidity encountered in this disease may occur due to lack of suspicion of this disease. In the present study, there was a five-day delay on average until adequate treatment was begun. An association of insufficient knowledge of the physiopathology and surgical anatomy of cervical deep spaces may delay diagnosis and effective treatment. The most frequently reported clinical symptoms in the literature correspond to the ones found in the present study, such as pain (89.2%), increased cervical volume (87.7%) and fever (75.4%).^14,35,37^ The clinician must not wait for fluctuation to make a diagnosis of deep neck infection, because this sign is rarely present. It needs to be borne in mind that these infections are deep, rather than superficial. Most patients had already sought medical attention and nearly half of them were using inadequate antibacterial treatment (47%), thereby reinforcing the impression of a lack of clinical suspicion and consequent delayed diagnosis.

The neck space most frequently described as affected by deep neck infection is the submandibular space.^1,3,4,10,14,31,33,40^ However, in our study the lateropharyngeal space was the space most frequently affected, according to computed tomography. The submandibular space was the site most frequently affected according to clinical findings, but was the second most frequent according to computed tomography. This is explained by the difficulty of inspection and palpation of the lateropharyngeal space, which is a deep cervical space.

Computed tomography scan is the most important imaging examination for correct evaluation of neck spaces affected by deep neck infections.^9,11,15,34-36,43,44^ Its importance lies in enabling determination of the correct surgical approach towards such patients, since in most cases there is more than one cervical space affected. Computed tomography scan presented good effectiveness in determining all the sites involved in this disease, in comparison with surgical findings.

The spread of the disease could be demonstrated by the extent of swelling or contrast enhancement of the soft neck tissues in the computed tomography scan.

A chest X-ray is also essential in determining therapeutic planning, because it may indicate more severe complications, such as mediastinitis and pleural effusion.^11,18^ The chest X-ray was altered in 6.3% of our patients. In our experience, when neck swelling is observed to reach the suprasternal notch, it is recommendable to extend the computed tomography scan from the neck to the mediastinum, in order to evaluate the spread of the infection.

*Streptococcus and Staphylococcus* are the most frequent findings of microbiological agents reported in literature, as well as in our patients.^3,13,14,17,23,24,31,37,45-47^ Other agents have clinical importance, especially anaerobes.^17,22,25,26,30,31^ The presence of more than one agent is quite common, ranging from 50 to 88% in the literature, but only 33% in our subjects.

Antibiotics and surgical drainage play a leading role in the treatment of deep neck infection. The initial recommended antibiotic is crystalline penicillin. Other valuable alternatives are first-generation cephalosporin or clindamycin. Other antibiotics may be necessary, as single agents or in association with the ones already mentioned, according to the bacterial culture and antibiogram. Other alternatives to drainage have been proposed and proven useful in some reports, such as direct or image-guided puncture and aspiration. In the present study, four patients were submitted to puncture unsuccessfully and all of these were definitively treated using surgical drainage a few days after the initial treatment. We consider surgical drainage to be the safest and most efficient alternative. A large incision permits expansion of tissue, thereby reducing compartment pressure, and this may be critical in preventing extension of the infection from one space to adjacent spaces. This allows better tissue oxygenation, with reduction of the anaerobic flora. The surgical approach can also assure upper airway patency in cases of actual or imminent upper respiratory distress.

The complications from deep neck infections have been decreasing, but high rates of morbidity and mortality still occur.^1,2,4-10,16,19-21,24^ The prevalence of complications shown in our data (26%) can be considered low and comparable to the literature.^1,2,5,6,10,14^ The death rate (7.7%) in our data is also lower than the findings reported in the literature.^2,16^ This can be explained by the early radical drainage techniques performed on our patients, as soon as the diagnosis of deep neck infection was made. Dissection of all of the spaces compromised by the disease that are represented by swelling on the computed tomography, and wound maintenance using drainage catheters until control of the disease is achieved, also plays an important role in the management of this serious infection in the head and neck area.

## CONCLUSIONS

Cervical pain, cervical mass, fever, odynophagia, locally increased temperature and dysphagia were the most frequent symptoms associated with deep neck infections. Dental manipulation and pharyngotonsillites were the most common etiological factors related to deep neck infection.

The most frequent physical findings were neck swelling, pain on palpation, local erythema and increased temperature. Clinical evaluation alone underestimated the extent of deep neck infection in 70% of the patients. Physical examination correctly evaluated the extent of infection in 30% of patients. Computed tomography scan correctly evaluated the extent of infection in all patients.

The most affected site via physical evaluation was the submandibular triangle, in 49.2% of the cases, but via computed tomography scan it was the lateropharyngeal space, in 65% of cases. More than one deep neck space was compromised in 90% of cases, as observed via computed tomography scan. Tissue swelling was the most important indicator of infection observed via computed tomography scan.
